# Corrosion Behavior of Niobium-Coated 316L Stainless Steels as Metal Bipolar Plates for Polymer Electrolyte Membrane Fuel Cells

**DOI:** 10.3390/ma14174972

**Published:** 2021-08-31

**Authors:** Yu-Sung Kim, In-Sik Lee, Jin-Young Choi, Shinhee Jun, Daeil Kim, Byung-Chul Cha, Dae-Wook Kim

**Affiliations:** 1 Advanced Manufacturing Process R&D Group, Ulsan Regional Division, Korea Institute of Industrial Technology (KITECH), 55 Jongga-ro, Jung-gu, Ulsan 44313, Korea; yskim80@kitech.re.kr (Y.-S.K.); lis0506@kitech.re.kr (I.-S.L.); choijy@kitech.re.kr (J.-Y.C.); 2 Total Marine Engineering Co., Ltd., 6 Goneul-ro, Dong-gu, Ulsan 44056, Korea; unknownbird@naver.com; 3 School of Materials Science & Engineering, University of Ulsan, 55-12 Techno Saneop-ro, Nam-gu, Ulsan 44776, Korea; dkim84@ulsan.ac.kr

**Keywords:** niobium, bipolar plate, corrosion resistance, fuel cell

## Abstract

Niobium was coated on 316L stainless steel by pulsed direct-current (DC) magnetron sputtering to improve corrosion behavior. The applied bias voltage highly affected the microstructure and crystallographic features, which lead to improved corrosion behavior. Due to the increased bias voltage, the microstructure of the niobium coating layer presented a smaller crystallite size and a densified structure, which obviously reduced the number of pinholes in the coated layer. Additionally, an increase in the degree of orientation toward the (110) plane, the most densely packed plane, lead to reduced dissolution of metal ions. Therefore, a pure niobium coating layer effectively protected the metal bipolar plate from a highly corrosive environment of polymer electrolyte membrane fuel cell (PEMFC) stacks. In particular, higher bias voltages of 600 and 800 V induced improved corrosion resistance, which satisfied the demand for the bipolar plate.

## 1. Introduction

Polymer electrolyte membrane fuel cells (PEMFCs) are one of the most promising alternatives to replace the internal combustion engine for the automotive industry. Although various automobile companies currently produce PEMFC stacks, several challenges remain to overcome the technical issues, such as a reduction in production costs, stack volume, and weight. The PEMFC stacks are composed of a membrane electrode assembly (MEA), a bipolar plate, and a gas diffusion layer (GDL). Among these components, the bipolar plate accounts for about 45% of the stack cost and 80% of the total weight [1,2,3]. The bipolar plate should be designed to accomplish several key functions. The roles of the bipolar plate are as follows: (1) to act as an electrical connector between each unit cells, (2) to act as a separator between fuel and the oxidizer, (3) to act as a structure supporter for stacks, (4) and to manage water for the membrane. To satisfy these functions, it needs to possess corrosion resistance in an acidic environment, excellent mechanical properties, high electrical conductivity, and gas impermeability [4,5].

Graphite and graphite-based composites have been widely used for the bipolar plate due to their excellent chemical stability and good electrical conductivity. However, a lower mechanical endurance of graphite causes lower workability, which leads to a cost increase in mass production [6,7]. Therefore, a metal bipolar plate with higher mechanical properties, electrical conductivity, and workability could be an alternative candidate for mass production.

Stainless steels can be considered as materials for bipolar plates due to lower costs and superior workability. These properties facilitate mass production and the fabrication of a very thin plate. Among stainless steels, austenitic stainless steel possesses the best corrosion resistance and workability. Hence, a significant amount of researchers’ efforts has been put into developing metal bipolar plates using austenitic stainless steel [8,9,10,11]. However, during long-term operation, the highly corrosive environment of PEMFC stacks leads to the dissolution of metal ions (e.g., Cr^2+^ and Ni^2+^) at the metal surface, which can be contaminated by electrolytes and catalysts. Consequently, the degradation of power density is accelerated. Therefore, surface modification is essential for developing the metal bipolar plate.

In order to protect the metal bipolar plate, various methods have been researched. Due to the thin and uniform layers, physical vapor deposition techniques have been widely applied. To improve corrosion resistance in an acidic PEMFC environment, nitride, alloy, and composite coatings were investigated [12,13,14,15], but electrical conductivity was relatively degraded compared to pure metals. Since niobium (Nb) has a superior chemical stability and corrosion resistance compared to other materials, it could be used as a material for the bipolar plate. In particular, it possesses higher corrosion resistance in sulfuric acid media, which is the main component of the electrolyte for PEMFCs. According to previous reports, cladded pure Nb presented no weight loss in sulfuric acid with 2 ppm HF for 2000 h, and lower interfacial contact resistance [16,17]. However, during the annealing process under a high temperature, a brittle intermetallic layer was formed between the substrate and the cladding layer [18], which might cause fracturing and resistance. Therefore, in this work, the Nb is coated on the metal substrate. Recently, pulsed direct-current (DC) magnetron sputtering has been widely used due to several advantages, such as better arc control and higher plasma density, as well as stability, than DC power. In order to effectively protect the substrate from corrosion, high-quality films are required, i.e., dense, uniform, and with fewer defects. Therefore, the Nb coating layers on the 316L stainless steels (SS316L) were investigated by the pulsed DC magnetron sputtering process. The morphologies and crystallographic features of the Nb coating layers due to bias voltage changes were characterized, and their effect on the corrosion resistance was investigated.

## 2. Materials and Methods

The cold-rolled SS316L sheet (Posco, Pohang, Korea) with a thickness of 0.1 mm was used as the substrate, and the chemical composition of SS316L is displayed in Table 1. The vacuum chamber for the pulsed DC magnetron sputtering (Infovion, Bucheon, Korea) has a cylindrical shape with a diameter of 500 mm and a height of 600 mm. The experimental conditions for sputtering are summarized in Table 2. The chamber was initially vacuumed and stabilized at 0.001 Pa. The specimen surface was then cleaned by plasma etching for 30 min at 0.666 Pa. Then, a ca. 1.5 μm thick Nb layer was deposited at the various bias voltages.

The electrochemical properties of Nb-coated 316L were investigated to examine corrosion behavior in response to different bias voltages. The polarization tests were performed using a typical three-electrode system. The saturated calomel electrode (SCE) was used as a reference electrode, and a platinum mesh was used as a counter electrode. The corrosion current and potential were measured by a potentiostat (Wonatech: WPG-100, Seoul, Korea). The samples were placed in a 70 °C, 0.5 M sulfuric acid (H_2_SO_4,_ Daejung Chemicals, Siheung, Korea) aqueous solution as an electrolyte while air and hydrogen were provided for cathode and anode conditions, respectively. The potentiodynamic test was conducted in the voltage range between −1.5 V to +1.5 V with a voltage scan rate of 5 mV/s to simulate the PEMFC operation conditions. To confirm electrochemical stability, a potentiostatic test was performed in both cathode and anode conditions. The current density was conducted at a potential of +0.6 V (cathode potential) and −0.1 V (anode potential) with provided air (cathode) and hydrogen (anode) for 3 h.

The surface and cross-section of Nb-coated layers were observed by a field emission scanning electron microscope (FE-SEM, JEOL JSM-5900LV, Tokyo, Japan) to examine microstructural characteristics. X-ray diffraction patterns were obtained using an X-ray diffractometer (Rigaku, D/Max 2500-V/PC, Tokyo, Japan) to examine the crystallographic features of and variations in the texture of the Nb-coated layers. To minimize the effect of the substrate, the glancing angle X-ray diffraction (GAXRD) mode was used. The XRD analysis was conducted using Cu Kα radiation over a 2θ range from 30 to 130° with a step size of 0.2° and a scan rate of 2°/min. The obtained data were calculated to investigate the crystallographic parameters.

## 3. Results

### 3.1. Electrochemical Characteristics

**Figure 1:**
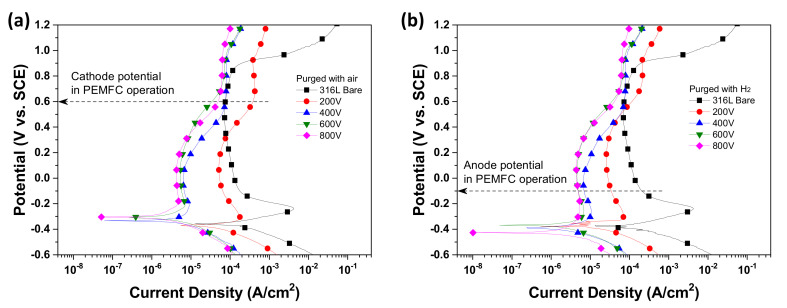
Potentiodynamic polarization curves of Nb-coated 316L samples in 0.5 M H_2_SO_4_ at 70 ℃ with purged (**a**) air and (**b**) H_2_.

In order to examine the effect of substrate bias voltage on the corrosion behavior of coated Nb layers, an electrochemical analysis was performed. All analyses were performed in the simulated PEMFC operating conditions for the cathode and anode, respectively. Detailed analysis conditions were explained in the method section. Figure 1 presents the potentiodynamic polarization curves of the Nb coatings deposited at the various bias voltages in the simulated PEMFC environment. Detailed parameters are summarized in the Table 3. In comparison with bare 316L at the cathode condition (Figure 1a), most of the Nb-coated 316L presented higher corrosion potential (*E*_corr_) than bare 316L. The *E*_corr_ tended to increase as applied bias voltage increased. In the case of the anode condition, the applied bias voltage of 600 V presented the highest *E*_corr_. The corrosion current density (*i*_corr_) that was obtained from the Tafel extrapolation method showed lower values than bare 316L at both the cathode and anode conditions. The current density in the PEMFC operation conditions for the cathode (*i*_0.6V_) and anode (*i*_−0.1V_) decreased with increasing bias voltage. In general, a lower value of critical passivation current density (*i*_crit_) and passivation current density (*i*_pass_) easily lead to a passivated state. The Nb-coated 316L presented lower values than bare 316L, and in particular, 600 and 800 V showed the lowest values. In the Tafel section, the anodic (*β*_a_) and cathodic (*β*_c_) Tafel slopes were determined from obtained polarization curves (Table 3). Generally, a *β*_a_ value for an anodic dissolution reaction is smaller than that of a *β*_c_ value for a cathodic reduction reaction in corrosive conditions [19,20]. Thus, a higher *β*_a_ than *β*_c_ value implies that it tends to passivate. If it corrodes, it has a higher *β*_c_ value. The determined Tafel slopes are as follows: The bare 316L and Nb layers coated at lower voltages (200 and 400 V) presented lower *β*_a_ than *β*_c_ values, while the higher *β*_a_ values of the Nb layers at the coated higher voltages (600 and 800 V) verify better corrosion behavior. The obtained results demonstrated that the coated Nb layer increases *E*_corr_ and decreases current density, including *i*_crit_ and *i*_pass_. As a result, the coated Nb layer effectively protects the surface of SS316L in the PEMFC environment.

The polarization resistance (*R_p_*) was calculated using the Stern–Geary equation as follows (Table 4):

(1) $$R_{p}=i_{\mathrm{corr}}\times \frac{\beta_{a}\times \beta_{c}}{2.303\left(\beta_{a}+\beta_{c}\right)}$$

The coated Nb layers increased the polarization resistance of bare 316L, and the polarization resistance tended to increase as the applied voltage increased. A higher polarization resistance means a higher tolerance to oxidation when an external potential is applied. As a result, the coated Nb layers at the higher applied voltages effectively protected bare 316L from corrosion in the aggressive oxidation condition. In addition, the corrosion rate (CR) was calculated using the following equation (Table 4):

(2) $$\mathrm{CR}=i_{\mathrm{corr}}\times \frac{K\times EW}{dA}$$

where *K* is a corrosion rate constant (3272), *EW* is the equivalent weight, *d* is the density of materials, and *A* is the exposed area of samples. In comparison with bare 316L, the coated Nb layers obviously decreased corrosion rate, which are one or two orders of magnitude smaller than that of bare 316L. In particular, the Nb layers coated at higher voltages presented the lowest corrosion rates. We assume that the improved *R_p_* and CR of Nb layers at the high voltages might be corresponding to less defects due to a densified microstructure, which effectively prevents contact with an aggressive electrolyte. This matter will be discussed further in the next section.

**Figure 2:**
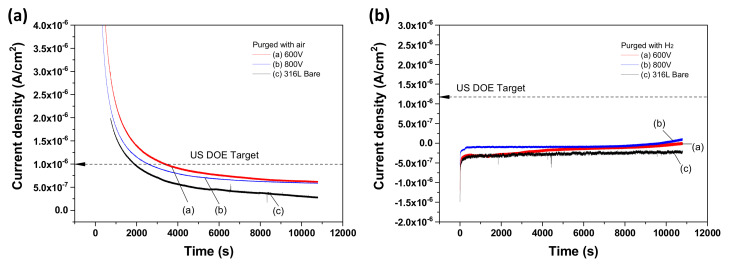
Potentiostatic polarization curves of Nb-coated 316L samples in 0.5 M H_2_SO_4_ at 70 ℃ (**a**) at 0.6 V with purged air and (**b**) at −0.1 V with purged H_2_.

In the operation of PEMFCs, the bipolar plate is exposed to both the cathode and anode in their operating voltages during long-term operation. The differences between free corrosion potential and operating voltages lead to corrosion at the surface [21]. Hence, the potentiostatic polarization test was performed in the simulated PEMFC environment for both the cathode and anode. Figure 2a presents polarization curves in the cathode environment. All of the samples presented a rapid current density drop at the initial stage and then the current density stabilized at a low value. A high current density at the initial stage might originate from the slower anodic oxidation reaction rather than the cathodic reduction reaction. After 2000 s, the reaction rate of anodic oxidation was about the same as the reaction rate of cathodic reduction due to charge conservation, and this equilibrium resulted in a low current density. At this time, the passive film began to form, and when it formed over the whole of the surface the current density was much lower. Thus, a rapid current density drop might be related to the formation of the passive film [21]. In the case of the anode environment (Figure 2b), the current density of samples was stabilized more quickly and the value was much smaller than in the cathode environment. These results imply the fast formation of the passive film and that it was cathodically protected with very slow dissolution. As a result, the Nb-coated samples accomplished the target of the US Department of Energy (DOE) in both the cathode and anode environments.

### 3.2. Microstructure

**Figure 3:**
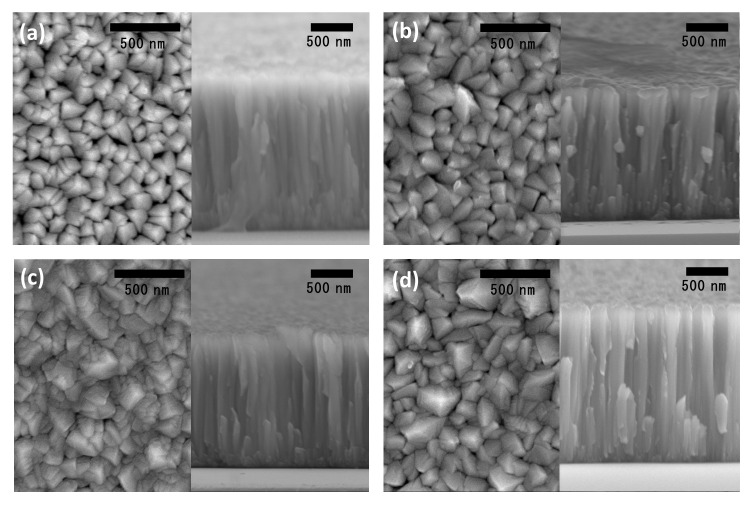
Surface and cross-section images of Nb-coated 316L at the various voltages: (**a**) 200 V, (**b**) 400 V, (**c**) 600 V, and (**d**) 800 V.

The morphology of Nb-coated samples at different bias voltages was observed using FE-SEM. As shown in the surface morphologies (Figure 3), the grain size was decreased and densified as a function of increasing bias voltage. In the case of the cross-sections, all samples presented a columnar structure including a void and grain boundary. Generally, the thin film deposited by physical vapor deposition without an interlayer has an open columnar structure of zone 1 in the Thornton structure model [22,23]. However, it is a subjective point of view to judge the density of the thin film by the outward eye. Furthermore, although the coated thin film has excellent corrosion resistance, the corrosion of the coated substrate might occur due to contact with the corrosive electrolyte through a defect on the surface and inside of the coated layer, such as a pinhole or a void. After the corrosive electrolyte runs through the substrate, it leads to electrical contact between the substrate and the coated layer, which results in galvanic corrosion. Consequently, the substrate is damaged and then the coated layer is peeled. Therefore, minimizing pinhole defects is important for protecting coating, and the pinhole defect of Nb coating was investigated. In order to examine pinhole defects, we used the critical passivation current density (CPCD) method, which electrochemically evaluates pinhole defects by measuring the polarization curve. The mechanism of the CPCD method is as follows: The *i*_crit_ of the metals involved in an activity–passivity transition increases in proportion to the surface area of metals. If the pinhole exists inside of the coated layer on the metals substrate and this pinhole is linked to the substrate, a part of the substrate that is exposed to a corrosive electrolyte can be dissolved. Thus, measured *i*_crit_ is proportional to the exposed surface area of the substrate [24,25,26]. The pinhole density (*δ*_pin_) is conducted using the following equation [27,28]:

(3) $$\delta_{\mathrm{pin}}=\frac{i_{\mathrm{crit}}\;\left(\mathrm{coated}\;\mathrm{sample}\right)}{2i_{\mathrm{crit}}\;\left(\mathrm{substrate}\right)}\times 100\%=\frac{i_{\mathrm{crit}}\;\left(\mathrm{Nb}/316L\right)}{2i_{\mathrm{crit}}\;\left(316L\right)}\times 100\%$$

where *i*_crit_ (316L) is the *i*_crit_ of bare 316L and *i*_crit_ (Nb/316L) is the *i*_crit_ of Nb-coated 316L. The constant in the denominator is the calibration factor, which assumes that the shape of the formed pit on the substrate is hemispherical [29,30,31].

**Figure 4:**
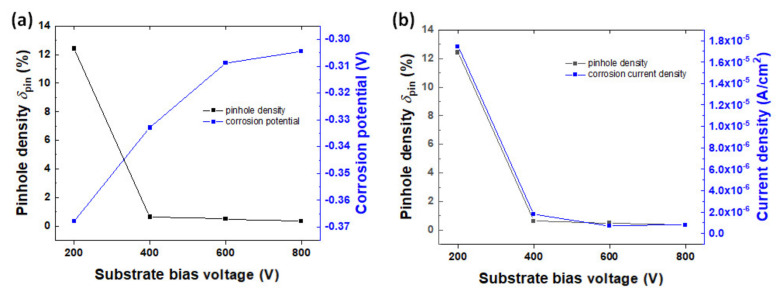
The effect of pinhole density variation on the corrosion (**a**) potential and (**b**) current density.

The calculated *δ*_pin_ is summarized in Table 5, and its relationship with corrosion potential and current density is presented in Figure 4. The calculated *δ*_pin_ tended to decrease as the bias voltage increased. Due to increased bias voltage, the microstructure of the coated layer was densified and the defect was decreased, which is consistent with FE-SEM observation. As explained above, the corrosion of coated metals’ substrates arises from contact with a corrosive electrolyte through a pinhole in the coating layer. Thus, a lower *δ*_pin_ means higher corrosion resistance. In fact, the 600 and 800 V samples presented improved corrosion behavior.

### 3.3. Phase Characterization

**Figure 5:**
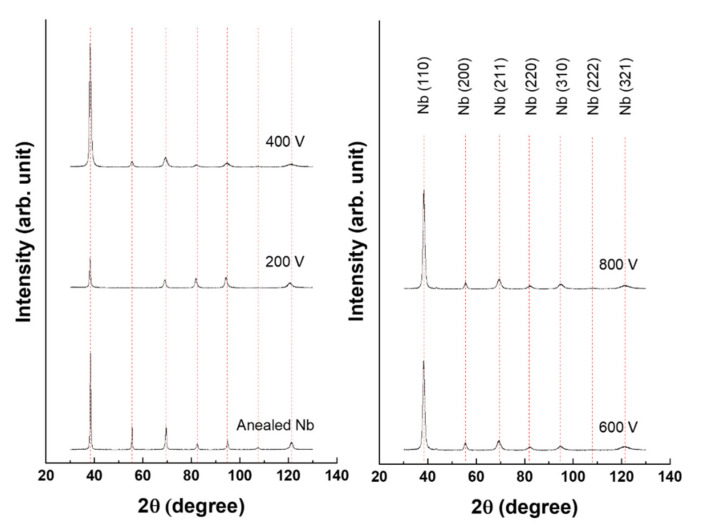
XRD patterns of Nb-coated 316L at the various voltages.

The XRD patterns of Nb-coated samples at the various bias voltages are shown in Figure 5. All samples presented a preferred orientation of (110). In the metal Nb with a body centered cubic (BCC) structure, the (110) plane is the most densely packed and has the lowest free surface energy. For this reason, the crystallite tends to grow toward the (110) plane that is perpendicular to the growth direction during deposition [32]. Thus, it showed a strong preferred orientation of (110). Altogether, the peak intensity of the (110) plane increased with increasing bias voltage. Furthermore, *d*-spacing was decreased and the integral width was increased due to increasing bias voltage (Table 6). The obtained results suggest that increased bias voltage leads to a smaller crystallite and denser microstructure.

**Figure 6:**
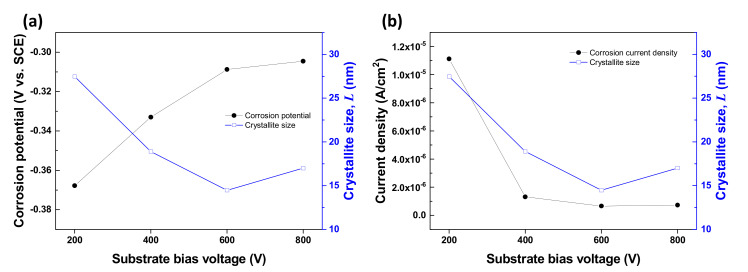
The effect of crystallite size variation on the corrosion (**a**) potential and (**b**) current density.

In order to understand the relationship between corrosion behavior and crystallographic features, we further characterized crystallographic parameters using the obtained XRD patterns. The obtained crystallographic parameters were summarized in Table 6. The crystallite size was obtained by the Williamson–Hall method [33,34,35]. As shown in Table 6 and Figure 6, the crystallite size decreased with increasing bias voltage. The increased bias voltage may cause stronger ion bombardment, which leads to the minimization of crystallite size. In the correlation between corrosion behavior and crystallite size (Figure 6), the obtained results suggest that the corrosion current density and corrosion potential improved as the crystallite size decreased. Generally, a smaller crystallite size induces an increase in the number of the grain boundary, which would lead to intergranular corrosion. However, there are several different points of view on this matter. As the crystallite size gets smaller, the diffusion rate of metal ions through the grain boundary is accelerated, which may cause an increase in the production rate of passive films. In addition, in the coated layer, although a smaller crystallite size generates a lot of grain boundary, densely deposited structures could reduce pinhole defects. In the present work, we assume that the reduced pinhole defect might be offset by the intergranular corrosion effect.

**Figure 7:**
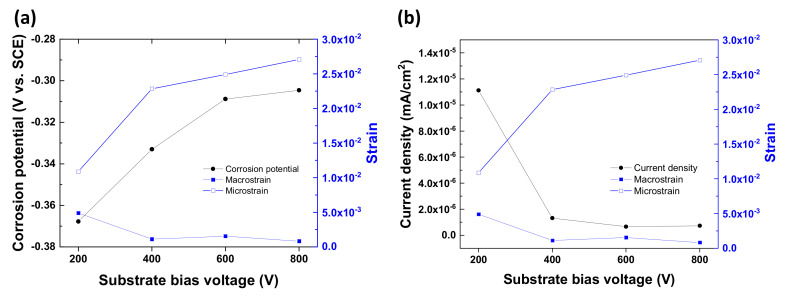
The effect of lattice strain on the corrosion (**a**) potential and (**b**) current density.

The effect of bias voltage on the strain of the coated layer was investigated. Figure 7 presents macrostrain and microstrain of Nb-coated SS316L, which were obtained by lattice variation and the Williamson–Hall method, respectively. As shown in the effect of strain on the corrosion behavior (Figure 7), the corrosion current density was reduced and the corrosion potential was increased due to an increase in macrostrain. In contrast, the microstrain variation presented an opposite result to that of the macrostrain. The increase of macrostrain arises from elastic deformation and leads to lattice expansion, which causes increasing residual stress. Subsequently, the increased residual stress negatively affects the corrosion behavior [36,37]. This increased residual stress could be relaxed due to plastic deformation related to microstrain. Thus, from the obtained results, the smaller macrostrain and the larger microstrain induce improved corrosion resistance.

**Figure 8:**
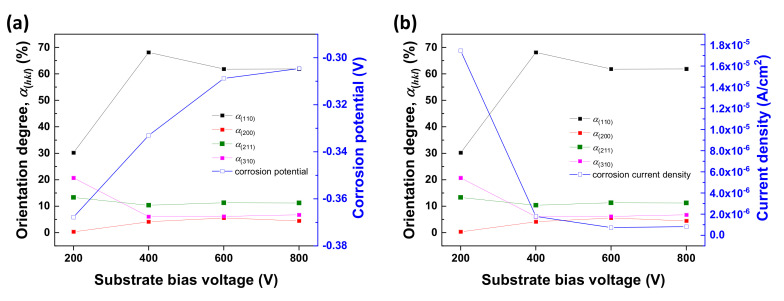
The texture variation due to bias voltage, and its effect on the corrosion (**a**) potential and (**b**) current density.

To examine the texture variation of Nb-coated 316L, the degree of orientation (*α_(hkl)_*) was calculated. In Figure 8, the degree of orientation toward the (110) plane increased as bias voltage increased. This means that the homogeneous Nb-coated layer is preferentially oriented toward the (110) plane due to a higher bias voltage. The texture variation of Nb-coated 316L indicated that corrosion resistance was improved with an increasing ratio of (110) texture. Because the (110) plane is the most densely packed in metal Nb with a BCC structure, it has stronger atomic binding energy than other planes of lower packing density. Thus, the dissolution of metal ions in the corrosive environment was inhibited. The obtained results suggest that the texture variation, due to bias voltage, affects corrosion behavior significantly. The increased bias voltage induces a strong texture for the (110) plane, which contributed to improved corrosion resistance.

Based on the above results, the coated Nb layers improved the corrosion resistance of metal substrates in the PEMFC operation conditions, but it could not completely protect SS316L due to a few defects including pinholes and voids. Although some defects still remain in the films, a pinhole density reduced to 0.33% and the investigation other process parameters could further improve the density of the films. Moreover, not only protecting films but modifying substrates would also improve corrosion resistance. In our previous work, nitrogen ions were implanted into SS316L, which affected corrosion behavior positively, and thus a continuous process from substrate modification to protective coating could lead to further improved corrosion resistance.

## 4. Conclusions

The pure Nb was coated on SS316L at the various bias voltages, and the Nb-coated layer improved the corrosion behavior of the substrate. The applied bias voltage highly affected the microstructure and crystallographic features, which lead to improved corrosion behavior. The increasing bias voltage resulted in smaller crystallite size and a densified microstructure. Corrosion potential was improved by ca. 0.3 V, and polarization resistance and corrosion rate also improved by more than 100 and 50 times, respectively, at the high voltages. In the CPCD analysis, the densified microstructure reduced pinhole density from ca. 12.4% to ca. 0.33%, which protected the substrate from contact with corrosive electrolytes. Moreover, due to increasing bias voltage, the (110) texture was increased, which inhibited the dissolution of metal ions in the corrosive environment. Therefore, as a corrosion-resistive layer for the metal bipolar plate, pure Nb coating was effective, and these results could provide experimental evidence of crystallographic effects for the development of metal bipolar plates for PEMFCs.

## Figures and Tables

**Table 1:** Chemical composition of 316L stainless steel.

Elements	Cr	Ni	Mn	Si	C	Mo	S	N	Ti	B	Cu	Fe
Wt%	16.62	10.1	1.35	0.43	0.018	2.06	0.028	0.046	0.01	0.01	0.34	Balance

**Table 2:** Experimental conditions for Nb sputtering.

**Constant**	Pressure (Pa)	0.666
	Gas flow (sccm)	10
	Target power (W)	60
	Working time (min)	40
**Variables**	Bias voltage (V)	200, 400, 600, and 800

**Table 3:** Corrosion parameters extracted from potentiodynamic polarization curves.

Sample	*E*_corr_ (V)	*i*_corr_ (A/cm^2^)	*i*_0.6V vs. SCE_ (A/cm^2^)	*i*_−0.1V vs. SCE_ (A/cm^2^)	*i*_crit_ (A/cm^2^)	*i*_pass_ (A/cm^2^)	*β*_a_ (V)	*β*_c_ (V)
316L bare	−0.3374	1.33 × 10^−4^	4.95 × 10^−5^	1.31 × 10^−4^	7.26 × 10^−4^	3.64 × 10^−5^	0.0416	0.0545
200 V	−0.3678	1.11 × 10^−5^	3.84 × 10^−4^	3.40 × 10^−5^	1.80 × 10^−4^	5.11 × 10^−5^	0.0211	0.0335
400 V	−0.3330	1.31 × 10^−6^	7.54 × 10^−5^	6.88 × 10^−6^	9.19 × 10^−6^	6.21 × 10^−6^	0.0459	0.0321
600 V	−0.3088	6.59 × 10^−7^	3.44 × 10^−5^	4.95 × 10^−6^	7.16 × 10^−6^	5.25 × 10^−6^	0.0657	0.0548
800 V	−0.3046	7.26 × 10^−7^	4.87 × 10^−5^	4.83 × 10^−6^	4.91 × 10^−6^	4.17 × 10^−6^	0.0497	0.0410

**Table 4:** Polarization resistance and corrosion rate calculated from potentiodynamic polarization curves.

Parameter	316L Bare	200 V	400 V	600 V	800 V
*R_p_* (Ω cm^2^)	61	322	4586	7045	11,927
CR (mmpy)	1.3903	0.4021	0.0474	0.0238	0.0263

**Table 5:** Pinhole density of coated Nb layers at the various applied voltages.

Pinhole Density	200 V	400 V	600 V	800 V
*δ*_pin_ (%)	12.41	0.63	0.49	0.33

**Table 6:** Crystallographic parameters of Nb-coated 316L at the various voltages.

Parameter	Annealed Nb	200 V	400 V	600 V	800 V
*d*-spacing, (Å)	2.344	2.360	2.353	2.342	2.342
Integral width, *β*	0.009	0.013	0.018	0.021	0.020
Crystallite size, *L* (nm)	165.593	27.461	18.902	14.478	17.004
Microstrain, η	0	1.088 × 10^−2^	2.284 × 10^−2^	2.491 × 10^−2^	2.708 × 10^−2^
Macrostrain, ε_φψ_	0	4.895 × 10^−3^	1.120 × 10^−3^	1.546 × 10^−3^	8.203 × 10^−4^

## Data Availability

The data can be provided by authors on request.
